# Translational proteomic study to address host protein changes during aspergillosis

**DOI:** 10.1371/journal.pone.0200843

**Published:** 2018-07-24

**Authors:** Guillaume Desoubeaux, David CHAUVIN, Maria del Carmen Piqueras, Ellen BRONSON, Sanjoy K. BHATTACHARYA, Gayle SIRPENSKI, Eric BAILLY, Carolyn CRAY

**Affiliations:** 1 University of Miami, Division of Comparative Pathology, Department of Pathology & Laboratory Medicine, Miller School of Medicine, Miami, FL, United States of America; 2 CHU de Tours, Parasitologie, Mycologie, Médecine tropicale, Tours, France; 3 Université de Tours, CEPR—INSERM U1100 / Équipe 3, Faculté de Médecine, Tours, France; 4 University of Miami, Mass Spectrometry Core Facility, Miller School of Medicine, Miami, FL, United States of America; 5 Maryland Zoo in Baltimore, Baltimore, MD, United States of America; 6 Mystic Aquarium, Mystic, CT, United States of America; Leibniz-Institut fur Naturstoff-Forschung und Infektionsbiologie eV Hans-Knoll-Institut, GERMANY

## Abstract

Aspergillosis is a fungal disease due to *Aspergillus* molds that can affect both humans and animals. As routine diagnosis remains difficult, improvement of basic knowledge with respect to its pathophysiology is critical to search for new biomarkers of infection and new therapeutic targets. Large-scale proteomics allows assessment of protein changes during various disease processes. In the present study, mass spectrometry iTRAQ^®^ (isobaric tags for relative and absolute quantitation) protocol was used for direct identification and relative quantitation of host proteins in diseased fluids and tissues collected from an experimental rat model challenged with *Aspergillus*, as well as in blood obtained from naturally-infected penguins. In all, mass spectrometry analysis revealed that proteome during aspergillosis was mostly represented by proteins that usually express role in metabolic processes and biological process regulation. Ten and 17 proteins were significantly ≥4.0-fold overrepresented in blood of *Aspergillus*-diseased rats and penguins, respectively, while five and 39 were negatively ≥4.0-fold depleted within the same samples. In rat lungs, 33 proteins were identified with positive or negative relative changes *versus* controls and were quite different from those identified in the blood. Except for some zinc finger proteins, kinases, and histone transferases, and while three pathways were common (Wnt, cadherin and FGF), great inter-species variabilities were observed regarding the identity of the differentially-represented proteins. Thus, this finding confirmed how difficult it is to define a unique biomarker of infection. iTRAQ^®^ protocol appears as a convenient proteomic tool that is greatly suited to *ex vivo* exploratory studies and should be considered as preliminary step before validation of new diagnostic markers and new therapeutic targets in humans.

## Introduction

Aspergillosis is a fungal airborne infection due to saprophytic ubiquitous molds that belong to *Aspergillus* genus [1]. It is responsible for several distinct respiratory diseases in both animals and humans. For example, aspergillosis is subacute or chronic in penguins under human care in zoos [2], and its incidence was estimated to 20.2% in a rehabilitation center in Brazil [3]. In such birds, it implies adaptive immunity and develops progressively at the inner surface of air sacs [2]. In humans, the invasive form is rather encountered during severe neutropenia and is more acute; its incidence raised to ≥5% after intensive chemotherapy or allogenic hematopoietic stem-cell transplantation [4]. Invasive aspergillosis causes high morbidity, and mortality rates have been estimated as 30–70%, depending on the underlying medical conditions, the site of infection and the degree of dissemination [5]. The *Aspergillus* species of the *Fumigati* section are those which have been primarily isolated from most of the human and animal clinical specimens. Species belonging to *Flavi*, *Nigri* or *Terrei* sections have been less frequently cultured [1].

Therapeutic failures during aspergillosis are partly due to the delay for establishing an accurate diagnosis, especially since the current laboratory diagnostic options are not numerous and display several limitations [1]. Detection of galactomannan antigen, *i*.*e*. a cell wall polysaccharide produced during hyphal growth, in patient blood by ELISA assay has made a major contribution to the diagnosis in the last ten years. It is now considered as a pivotal mycological parameter to achieve diagnosis of aspergillosis [6], but it sometimes suffers from a poor specificity in cases of concomitant administration of β-lactam antibiotics or polyvalent immunoglobulins [7]. Furthermore, the galactomannan test may be falsely negative in patients from clinical departments other than haematology: in a French cohort reporting 424 cases of aspergillosis, 68.7% and 69.1% serum samples were positive for individuals with acute leukaemia or allogenic hematopoietic stem-cell transplantation, *versus* 25.8% and 36.4% for patients with solid-organ transplantation or systematic inflammatory diseases, respectively (*P*<0.001) [5]. Furthermore in veterinary medicine, detection of galactomannan is not reliable at all in some birds, especially in raptors or parrots [8]. (1–3)-β-D-glucan, *i*.*e*. a fungal cell wall component, which has been recently incorporated into the European Organization for Research and Treatment of Cancer / Mycoses Study Group (EORTC/MSG) mycological criteria for invasive aspergillosis diagnosis in human patients, is a serum pan-fungal biomarker with a high negative predictive value, but with no specificity for *Aspergillus* genus [6]. The *Aspergillus*-specific lateral flow device, which is based on the JF5 antibody, detects an extracellular glycoprotein antigen secreted during active growth of *Aspergillus* spp. This point-of-care device permits rapid testing of easily obtainable specimens and has shown promising results in various studies, but it still needs further validation steps [9]. Quantitative polymerase chain reaction (qPCR) has not been standardized yet, and thus is nowadays not considered as a pivotal diagnostic criterion, although its potential benefit has been several times underlined [10]. Colony-forming unit (CFU) counting based on *in vitro* fungal cultures is not reliable enough to estimate the actual *Aspergillus* burden, because it does not reflect the total amount of viable, dormant and dead fungus within the tissue or the fluid that is investigated [1,11]. Detection of anti-*Aspergillus* antibody is not recommended in highly immunocompromised patients and is rather reserved for the indirect diagnosis of chronic aspergillosis [12]. It is systematically positive in penguin blood whatever the clinical status [13]. As alternatives, new biomarkers of aspergillosis are therefore needed, and it is likely that their definition will occur through the improvement of basic knowledge about pathophysiology. Study of the specific host response during aspergillosis may be able to provide such information.

In last decade, transcriptomics and proteomics have been largely used for addressing significant protein changes within various diseased organisms or pathologic fluids [14]. However, transcriptome profiling has limitations, because mRNAs and levels of corresponding proteins are not systematically linked due to post-translational modifications [15]. In addition to their capacity for accurate peptide characterization, some innovative mass spectrometry (MS) tools are now able to directly quantitate the relative amount of proteins identified within fluid or tissue, regardless if they are or are not post-translationally modified. For instance, the iTRAQ^®^ (isobaric tags for relative and absolute quantitation) protocol is a large-scale isobaric labeling method of unbiased profiling to identify and to determine the change in levels of proteins from different multiplexed sources within a single experimental run [16]. While generating no interference with peptide mass determination, iTRAQ^®^ technique uses stable isotope-tagged molecules that can be covalently bonded to the N-terminus and side chain amines of trypsin-digested peptides [17]. It requires no preliminary labor-intensive steps of protein separation, like two-dimensional gel (2D-gel) electrophoresis, that inevitably implies a risk of information loss [14,18], and which is less sensitive [18]. Moreover, iTRAQ^®^ protocol allows addressing protein modifications including those induced during pathologic processes leading to the formation of post-translationally modified proteins, as well as proteolytic fragments that may be pathogenic for tissue-invasive disease such aspergillosis.

Through application based on iTRAQ^®^ protocol, the goal of this study was to bring new insights into host protein responses against *Aspergillus*, using an experimental murine model of aspergillosis, as well as samples obtained from naturally-infected birds. The overall objective relies in investigating the pathophysiology of aspergillosis in order to suggest possible host biomarkers of infection, so that later facilitating diagnosis in birds, for which there are no adequate diagnostic assays, and perhaps to widen similar perspectives in humans for which rapid diagnosis is still sub-optimal to date. We are also searching for new therapeutic targets to enhance the anti-*Aspergillus* response.

## Material and methods

### Study population: Animal models of infection

#### Inclusion and samples

Blood samples and lung parenchyma specimens were obtained from 6–8 week-old male neutropenic rats (*Rattus norvegicus*, *N*_*1*_ = 46). Previously, rats had been immunocompromised by intraperitoneal injections of cyclophosphamide (Endoxan 1000^®^, Baxter, Guyancourt, France) repeated every four days, and had been intra-tracheally challenged with either 1 mg/kg bacterial lipopolysaccharides (*Salmonella enterica* Serovar Typhimurium 2 mg/mL LPS, Sigma-Aldrich, Saint-Quentin-Fallavier, France) (*n*_*1*_ = 14) or with 1.0x10^6^ *A*. *fumigatus* conidia (strain No. BRFM 1827, collection WFCC-MIRCEN *World Data Centre for Microorganisms*, Marseille, France) (*n*_*1’*_ = 32), according to the experimental model previously described [19,20]. The animals had free access to sterile water supplemented with 750 mg/L tetracycline antibiotic (Sigma-Aldrich, Saint-Louis, MO, U.S.A.) to prevent possible opportunistic bacterial infections, and with 300 mg/L acetaminophen (Efferalgan 150^®^, Bristol-Myers-Squibb, New York, NY, U.S.A.) as a painkiller.

Furthermore, routine blood samples were collected into BD PST 0.5 mL-Microtainer^®^ tubes (Becton-Dickinson, Le Pont-de-Claix, France) from African penguins (*Spheniscus demersus*, *N*_*2*_ = 112) under human care in seven facilities in the United States of America (U.S.A.).

Upon arrival to the laboratory in charge of the mass spectrometry experiment (University of Miami, FL, U.S.A.), all blood samples were centrifuged for 10 minutes (min) at 3000 *g* and stored at -80°C before being utilized for testing. The whole lungs were placed in sterile containers and flash frozen in liquid nitrogen before protein extraction.

#### Case definitions

For the rat protocol, blood sampling at baseline, *i*.*e*. before any tracheal challenge, defined the healthy status (blood collection by puncture of the lingual vein into 1.5mL-serum tubes). Rats inoculated with LPS were considered as inflammatory controls. For the rats that were experimentally-inoculated with *A*. *fumigatus* conidia, the diagnosis of “proven” aspergillosis was established when fungal elements were observed on histopathological slides of lungs prepared at date of death or at time of sacrifice [19]. The latter was decided when rats began to present deleterious clinical signs consistent with the development of aspergillosis, *i*.*e*. according to a discomfort scale which was checked thrice daily and scored from 1 to 6 on the basis of appearance changes (*e*.*g*. dirty nose, red-rimmed eyes, ruffled fur, extreme pallor), behavior changes (*e*.*g*. gasping, wheezing, prostration, instability), reaction to stimuli and variation of body weight,… Score 1 was consistent with no discomfort; score 2, minor discomfort; score 3, poor discomfort; score 4, serious discomfort; score 5, severe discomfort; and score 6, death. Score 3 was set as the endpoint; it encompassed respiratory troubles, prostration, bleeding,… [21]. For control rats, the sacrifice was scheduled one to two days after LPS instillation, when the inflammatory reaction was assumed to be maximal [14]. Practically, rats were killed after anesthesia with 5% isoflurane (Aeranne^®^, Baxter, Deerfield, IL, U.S.A.) by the intraperitoneal injection of 0.5 mL of 2.5% thiopental (Nesdonal^®^, Sanofi-Aventis, Montrouge, France). In cases histopathology was non-contributive, the diagnosis of “probable” aspergillosis was retained in rats when were present at least two positive biomarkers among the following ones: galactomannan antigen in the blood with index > 0.5 in accordance with the kit manufacturer’s instructions (EIA Platelia *Aspergillus*^®^, Bio-Rad, Marnes-la-Coquette, France) (index is defined as the ratio of the optical density value of the sample to the value of a standard containing 1 ng of galactomannan); galactomannan antigen in the bronchial-alveolar lavage fluid (BALF) with index > 1.0; or 48h fungal culture with ≥ 5 CFU *per* 100 μL BALF pellet that had been inoculated onto Sabouraud-dextrose agar with gentamicin and chloramphenicol (ThermoFisher Scientific, Dardilly, France) [14]. If histopathology was negative and only one or less of the three abovementioned biomarkers was present in *Aspergillus*-challenged rats, the corresponding samples were considered as equivocal and then excluded from the subsequent analysis. According to the aforementioned restrictions, we were able to collect 18 blood samples at baseline, 12 after LPS-challenge and 14 after *Aspergillus*-challenge. Ten and 16 lung parenchyma specimens were also obtained from control and infected animals, respectively. Besides, there were no expected deaths due to other types of infection in this protocol, as confirmed by sterile cultures on media specifically dedicated to bacterial growth (chocolate agar PolyViteX®, BioMérieux, Craponne, France).

Aspergillosis classification in penguins was derived from the definition utilized in human medicine [6,10]. The definitive diagnosis of “proven” fungal infections was based upon histopathologic evidence [1]. The diagnosis of “probable” aspergillosis was retained upon positive fungal culture (from any tissues that were assumed to be relevant for mycological investigations, *i*.*e*. primarily lungs or air sacs) in conjunction with clinical examination findings (*e*.*g*. dyspnea, wheezing, gasping, stridor, open-mouth breathing, coughing, changes in vocalizations,. lethargy, anorexia, weight loss, …) or imaging investigations (celioscopy and/or X-radiography performed for 40.9% birds) and/or any medical records which corroborated the diagnosis. Since galactomannan antigen detection has been shown to be unreliable in birds, it was not considered herein to be a pivotal parameter for the diagnosis of “probable” aspergillosis in penguins [8,22]. Samples obtained from birds with clinical signs of aspergillosis, but with neither positive fungal cultures nor imaging findings available, remained in the study if the animal responded well to antifungal treatment and had detectable anti-*Aspergillus* antibodies in blood associated with marked changes in the protein electrophoretogram performed on the Spife 3000^®^ electrophoresis analyzer (Helena Labs, Beaumont, TX, U.S.A.) [13]. Detection of anti-*Aspergillus* antibodies was conducted *via* a previously described ELISA technique using bulk *Aspergillus* ID antigens made of pooled mycelial-phase culture filtrates of *Aspergillus* species (IMMY, Norman, OK, U.S.A.) [13]. These cases were therefore considered “possible” diagnosis. The samples obtained from penguins with equivocal context of aspergillosis were excluded. Animals with no apparent clinical signs were considered healthy controls, while those undergoing miscellaneous non-*Aspergillus*-related processes, including other infections, were classified as inflammatory controls. In all, 38 samples were collected from 34 diseased penguins at time of aspergillosis diagnosis (for two penguins, sampling were performed in triplicate). Fifteen follow-up samples were obtained for 14 of them, in average 55.6 ± 31.5 days after the initial diagnosis (and thus defined the “convalescent” group when clinical improvement was noticed and/or long-term antifungal therapy was given). Ninety-eight samples were drawn from 78 other penguins, including 53 healthy birds and 25 non*-Aspergillus* inflammatory controls.

### Proteomic analysis

#### Preliminary extraction of proteins from tissues

After thawing, rat lungs were manually crushed and soaked in 1% sodium dodecyl sulfate (SDS) extraction buffer and then homogenized for two min. Homogenates were centrifuged at 12,000 *g*, and the supernatants were boiled at 95°C for 5 min and neutralized with 100 mM Tris, pH10 during 10 min. Cold acetone was added and incubated on ice for 15 min. After centrifugation, the pellets were dried in an Eppendorf Vacufuge^®^ concentrator (ThermoFisher Scientific, Waltham, MA, U.S.A.), and re-suspended in 20 μL water and 10mM dithiothreitol. At that point, all the lung protein extracts were processed for proteomic study as with the blood samples described below.

#### Distribution into distinct aliquot groups

For each animal species, blood samples or tissue protein extracts were pooled into single tubes according to the medical group they belong to, by including 1000 μg protein for each in order to constitute distinct representative aliquots. For instance for the rat protocol, the five following representative groups were defined: in blood at baseline (*i*.*e*. before the experimental challenge), in blood after inflammation-challenge with LPS, in blood after *Aspergillus*-challenge, in lung parenchyma after LPS inflammation-challenge, in lung parenchyma after *Aspergillus*-challenge. For the penguins, the four following groups were constituted by pooling separately the blood samples for clinically-normal animals, non-*Aspergillus* inflammatory controls, *Aspergillus*-diseased animals, and convalescent subjects (see definition above).

#### Pre-processing step with iTRAQ^®^-tags labeling

Protein enrichment was completed in each of the pooling tubes using the Pierce Albumin / IgG removal^®^ kit (ThermoFisher Scientific, Waltham, MA, U.S.A.) that allows for depletion of overrepresented proteins like albumin or immunoglobulins [23], as demonstrated in previous works with mammal and chicken samples [14]. Total protein was quantified in each pool by the Pierce BCA Protein assay^®^ kit (ThermoFisher Scientific, Waltham, MA, U.S.A.), according to the manufacturer’s instructions [23]. Then, 100 μg of each pool was incubated with 30 μL dissolution buffer of 0.5 M triethylammonium bicarbonate (Sigma-Aldrich, Saint-Louis, MO, U.S.A.) at pH 8.5, and thereafter treated with 2% SDS-denaturant solution. One microliter of tris-(2-carboxyethyl)phosphine-reducing reagent was added, followed by vortex-shaking for 1 min, centrifugation at 18.000 x *g* for 5 min and incubation at 60°C for 1 h. After another step of shaking and centrifugation, 84 mM iodoacetamide was added for a 30 min-long incubation in the dark at room temperature. Freshly prepared 0.1 μg/μL sequencing grade modified trypsin was then mixed with each sample, and digestion was carried out at 37°C during 30 min. Thereafter, 1 μL trypsin was added to continue digestion overnight. The samples were dried in an Eppendorf Vacufuge^®^ concentrator, then reconstituted with 30 μL dissolution buffer. Every tube was mixed with one unique iTRAQ^®^ reagent vial (Sigma-Aldrich, Saint-Louis, MO, U.S.A.) and reconstituted in isopropanol, according to the manufacturer’s recommendations. For instance for rats, the tube containing all the blood samples pooled at baseline was mixed with the 113-iTRAQ^®^ reagent; this containing the blood samples from control rats undergoing LPS inflammation with the 114-reagent; this containing the blood specimens from *Aspergillus*-diseased rats with the 115-reagent; this containing the pool of lung protein extracts from LPS-control rats with the 116-tag; and the aliquot containing the pool of lung protein extracts from *Aspergillus*-diseased rats with the 117-reagent. Thereafter, the contents of all these iTRAQ^®^ reagents-labelled tubes were combined into one single tube *per* species, vortexed for 1 min, and centrifuged at 18,000 *g* for 5 min. The multiplexed specimen was totally dried by centrifugal vacuum concentration as above. The same procedure was separately applied for all of the penguin samples, according to the repartition into distinct clinical groups that were defined above.

#### Processing steps by mass spectrometry iTRAQ^®^ protocol

The aliquots of the two multiplexed iTRAQ^®^ tubes (one for all the rats and one for all the penguins,) were separately re-suspended in 2% acetonitrile and individually loaded onto an ultra-high performance liquid chromatographic (UHPLC) method. UHPLC analysis was conducted according to the manufacturer’s indications in an Easy Nano LC 1000^®^ model (ThermoFisher Scientific, Waltham, MA, U.S.A.), where a 75 μm i.d. x 15 cm column, packed with Acclaim PepMap^®^ RSLC C18-2 μm 100Å column (ThermoFisher Scientific, Waltham, MA, U.S.A.) was in-line connected to a Acclaim PepMap^®^ 100 75 um x 2 cm, nanoviper C18-3 μm 100 Å pre-column (ThermoFisher Scientific, Waltham, MA, U.S.A.). Peptides were eluted following a 75 min gradient from 2% to 98% acetonitrile (ThermoFisher Scientific, Waltham, MA, U.S.A.) in UHPLC water at a flow of 350 nL/min. Then, the eluates were individually run in a QExactive^®^ Orbitrap mass spectrometer (ThermoFisher Scientific, Pittsburg, PA, U.S.A.) in a data-dependent mode, with an automatic gain control target of 1.0x10^6^ for full MS at 70,000 resolution, and of 2.0x10^5^ for dd-MS^2^ at 17,500 resolution in positive mode. The detection performance of this QExactive^®^ Orbitrap were previously evaluated as follows: high resolving power of 140,000 full width at half maximum, defined at *m*/*z* 200, and elevated resolution at Δ_*m*/*z*_ = 0.001, with a 50–4,000 *m*/*z* range. The isolation window was fixed to 1.5 *m*/*z* with normalized collision energy of 28 eV, underfill ratio of 1.0%, and dynamic exclusion of 3.0. First mass was fixed to 103 *m/z*. Each aliquot was tested in triplicate.

#### Mass spectrometry data analysis and identification/quantitation of host proteins

The bioinformatics analysis was performed using the Proteome Discoverer^®^ software (ThermoFisher Scientific, Pittsburg, PA, U.S.A.). Specific SwissProt^®^ reviewed non-redundant databases (http://www.uniprot.org/uniprot/) were used for the analysis of rat (according to the following genera: *Rattus*, *Fukomys*, *Heterocephalus*, *Niviventer*, *Thryonomis*, *Ctenodactylus*, *Malacomys*, *Spalax*, *Ondrata*, and *Mus*) and penguin (*Spheniscus*, *Aptenodytes*, *Pygoscelis*) proteomes using the Sequest^®^ HT search engine (Washington, DC, U.S.A.) [24]. The parameters of the study set trypsin as the enzyme used for digestion, with a maximum number of missed cleavage sites of two, 10 ppm as precursor mass tolerance, and 0.02 Da for fragment mass tolerance. Alkylation, as well as N-terminal / lysine modifications and dynamic iTRAQ^®^ modifications were selected as fixed, monoisotopic mass was chosen, and threshold for expected ion score cut-off was inferior to 0.05, 95% confidence. Maximum value for delta-correlation was set to 0.05. False discovery rate target value was 0.01 for strict rate and 0.05 for relaxed one, considering the *q*-value as a validation reference. At least one unique peptide was necessary for each protein identified. Median intensities were used for normalization, and outliers were removed automatically. Search results were passed through additional filters to exclude unlabeled proteins before exporting the data. Protein identification was achieved using a Bayesian algorithm where matches were characterized by an expectation score, which represents an estimate of the number of matches that would be expected in that database [18].

At the quantification level, the analysis included Κ-means clustering, *i*.*e*. supervised and heuristic algorithm, for both protein and peptide quantitation ratios. The software actually worked by grouping proteins, based on the peptide spectral matches (PSMs). Then, a customized ratio was calculated for every protein group as the median of all PSMs included in the protein group. Only ratios with *P* < 0.05 and only fold-changes ≥ 2.0 across three aliquot replicates were used to determine up- or down-regulated proteins [25].

### Validation assays

In order to confirm some findings of the MS analysis, the levels of Wingless integration site-1 (Wnt1) inducible signaling pathway protein 1 (WISP-1) levels were measured in distinct blood samples of the rat model using the Rat WNT1 Inducible Signaling Pathway Protein 1 ELISA® kit (MyBioSource, San Diego, CA, U.S.A.) [26], according to the manufacturer’s instructions. Ten samples of each group of the protocol were tested simultaneously in duplicate.

The levels of basic fibroblast growth factor (bFGF) were tested in the supernatants of the lung homogenates by the means of the RayBio® Rat bFGF ELISA kit (RayBiotech, Norcross, GA, U.S.A.), tuning the manufacturer’s recommendations to that of cell culture media [27]. Ten samples of each group of the rat protocol were tested simultaneously in duplicate.

### Statistical analysis

Statistical analyses were performed using XLStat^®^ v.2016.6.04 software (Addinsoft, Paris, France). Missing data, *e*.*g*. when the total volume of sample was insufficient to complete all the analyses, were managed by the method of mean imputation. Mann-Whitney, Chi-squared and Kruskal-Wallis tests were applied. The α-risk was adjusted at 0.05.

Regarding specifically the iTRAQ^®^ data, we based inference on one-way analysis of variance (ANOVA) models, conducted on one protein at a time [28], combining both normalization, *i*.*e*. bias removal, and assessment of differential protein expression in a single model fit to the collection of reporter-ion peak areas, *i*.*e*. corrected for isotopic overlap, from all observed tandem mass spectra. Principal component analysis (PCA) modeling was applied to facilitate interpretation of the multivariate proteome dynamics dataset [29]. Data were z-scored prior to model building.

To perform enrichment analysis on gene sets that were found up- and down-regulated during aspergillosis, Gene Ontogeny (GO) base (http://www.geneontology.org/) was used for every model. The following species names were selected on GO base: *Rattus norvegicus* for the rats, and *Gallus gallus* for the penguins (because of no specific database available for any penguin genera). The annotation data set used for the bioinformatic analyses were extracted by overrepresentation test from PANTHER classification system version 11.1 (released 2016-10-24) [30]. Bonferroni correction was used for multiple testing during expression data analysis.

### Ethics

The authors applied the regulations of Declaration of Helsinki. They complied with the BRISQ guidelines.

The rat model of invasive aspergillosis was approved by the General Direction for Research and Innovation, French Ministry of Higher Education and Research through the accreditation number No. 01901.01, and by the Ethics Committee for Animal Experimentation of the Val-de-Loire region through the accreditation number No. C37-261-3 [14].

All the blood specimens from penguins utilized in this work were samples banked after routine veterinary assessments. Investigations in penguins were approved by the African Penguin Species Survival Plan (SSP) of the Association of Zoos & Aquariums (AZA) program and by the staff veterinarians in each of the submitting facilities.

Informed consent is not applicable.

## Results

### Description of animal and patient populations

In rats, the mean index for galactomannan antigen in blood was 0.1 ± 0.1 at baseline. After experimental inoculation with *Aspergillus*, the mean measure at time of death was 2.8 ± 2.9 in blood and 3.2 ± 2.4 in BALF, while histopathology was positive in 82.6% cases. In control rats, the mean measure for galactomannan antigen was estimated at 0.2 ± 0.1 in blood and 0.7 ± 0.4 in BALF, whereas histopathology observation was systematically negative for fungus. Fungal cultures showed an average of 28.4 ± 13.7 CFU/100 μL BALF pellet in *Aspergillus*-challenged rodents *versus* 0.0 ± 0.0 CFU in controls. Eight blood samples and three lung protein extracts were excluded from the analysis as they did not fulfill the inclusion conditions.

Characteristics of included penguins are detailed in Table 1. Measurement of galactomannan antigen in blood displayed the following mean values: 0.5 ± 1.3 and 0.3 ± 0.3 indexes for *Aspergillus*-diseased penguins and inflammatory controls, respectively. There were no differences for anti-*Aspergillus* antibody titers between cases and controls (*P*>0.05). In *Aspergillus*-diseased penguins, α2- and β-globulins were significantly increased *versus* controls (*P*<0.05), while albumin / globulin ratio was decreased (*P*<0.01). Eight penguin blood samples were rejected, because they did not meet the inclusion criteria.

**Table 1:** Characteristics of the penguins included in the study. The assignment was made according to the case definition for diagnosis of aspergillosis (for details, see *Material & Methods* section).

	Mean (± SD) or Number (%), [_95%_confidence interval]	
Study population of the penguin cohort (*N*_*2*_ = 112)	*Aspergillus*-diseased cases (*n*_*2*_ = 34)	Controls^ǂ^ (*n*_*2”*_ = 78)
**Age** (years)	**7.6** (± 7.1), [5.4–9.8]	**11.6** (± 9.9), [9.4–13.9]
**Sex** (male)	**21** (61.8%), [48.3–77.2%]	**40** (51.3%), [40.8–63.1%]
**Clinical signs**		
respiratory^a^	**23** (67.6%), [51.9–81.5%]	**9** (11.5%), [5.0–17.5%]
general^b^	**26** (76.5%), [63.7–90.1%]	**19** (24.4%), [16.0–33.0%]
neurological^c^	**4** (11.8%), [2.3–23.3%]	/
**Protein electrophoresis**^φ^		
albumin / globulin ratio	**0.4** (± 0.4), [0.3–0.6]	**0.8** (± 0.5), [0.7–0.9]
albumin (g/dL)	**1.4** (± 0.8), [1.0–1.8]	**2.0** (± 0.5), [1.9–2.1]
α1-globulins (g/dL)	**0.1** (± 0.0), [0.1–0.1]	**0.2** (± 0.1), [0.1–0.2]
α2-globulins (g/dL)	**1.2** (± 0.5), [1.0–1.5]	**0.8** (± 0.3), [0.6–0.9]
β-globulins (g/dL)	**1.8** (± 0.6), [1.5–2.0]	**1.6** (± 0.8), [1.2–1.8]
γ-globulins (g/dL)	**1.4** (± 0.8), [1.0–1.7]	**2.0** (± 1.5), [1.6–2.4]
**Findings for aspergillosis diagnosis**		
suggestive imaging	**11** (32.6%) [18.1–45.6%]	**2** (2.6%) [0.0–4.8%]
positive *in vitro* culture	**2** (5.9%) [0.0–14.3%]	/
anti-*Aspergillus* antibody ELISA optical density^φ^	**1.8** (0.3%) [1.6–1.9]	**1.9** (0.3%) [1.8–2.1]
positive histopathology observation	**5** (14.7%) [5.1–26.7%]	/
**Response to antifungal treatment** (positive)	**28** (82.6%) [68.5–93.7%]^$^	**/**

^**ǂ**^ included samples from clinically-normal penguins (*n* = 53) and from control cases undergoing arthritis (*n* = 6), anemia (*n* = 4), malaria (*n* = 3), carcinoma (*n* = 2), or other miscellaneous causes of inflammation (*n* = 10)

^a^ *i*.*e*. dyspnea, wheezing, gasping, stridor, open-mouth breathing, coughing, changes in vocalizations

^b^ *i*.*e*. lethargy, anorexia, weight loss, lack of appetite, sternal recumbency, regurgitation

^*c*^ *i*.*e*. ataxia, opistophonos, torticollis, limb paresis, blindness, behaviour changes

^φ^ according to Cray *et al*. [13]

^$^ two penguins were lost to follow up

### Proteomic analysis

**Fig 1:**
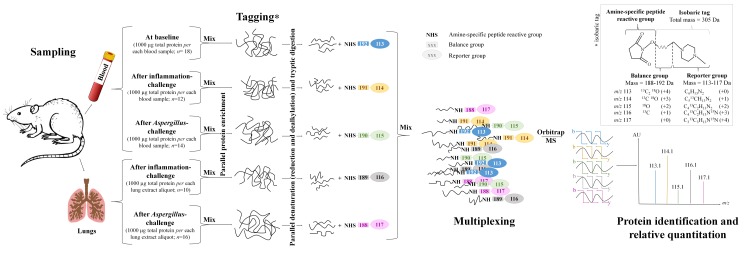
Example of study design for iTRAQ^®^ protocol in the experimental rat model of invasive aspergillosis. *Upper right panel*, the iTRAQ^®^ reagent is designed as an isobaric stable tag consisting in a charged reporter group that retains charge (N,N-dimethylpiperazine), a peptide reactive group (N-hydoxy-succinimide) that is amide-linked to the N-terminus and the ε-amino side chains of all the peptides got from prior tryptic digestion, and a neutral balance portion (carbonyl) to maintain an overall mass of 305 kDa by the means of differential isotopic enrichment with ^13^C, ^15^N and ^18^O atoms [33]. The selection of the reporter region in the low mass area enables keeping the additive mass to the fragments as negligible as possible in order to minimize any side effect during chromatographic separation and to avoid any interference with other fragment ions during mass spectrometry analysis, thus allowing for the highest degree of confidence; *Main panel*, after intra-tracheal challenge with bacterial lipopolysaccharides (LPS) or *Aspergillus fumigatus* conidia, rat samples were pooled according to their clinical status: healthy controls at baseline before intra-tracheal challenge in blood, non-*Aspergillus* inflammatory controls and *Aspergillus*-diseased cases, both in blood and in lung parenchyma for the last two groups [19,20]. Overabundant proteins, like albumin and immunoglobulins, were removed through a commercial kit before mass spectrometry analysis [23,56], and specific labelling with individuals tags, ranging from 113-iTRAQ^®^ to 117-iTRAQ^®^ reagents, was then applied to each pooled aliquot [16]. The reporter-balance peptides remained intact, so that for one given common protein, the five multiplexed rat samples had an identical *m/z*: the peptide fragments were equal, only the reporter ions were different. Indeed, the precursor ions, and all the internal fragment ions, *i*.*e*. type b- and y-ions respectively, contain all five members of the tag set, but remain isobaric, *i*.*e*. the five species have the same atomic mass but different arrangements. After collision in the QExactive^®^ Orbitrap mass spectrometry (MS) instrument, the five reporter group ions appeared as distinct masses ranging between *m/z* 113–117, while the remainder of the sequence-informative b- and–y ions remain isobaric and their individual current signal intensities were additive. The relative concentration of the peptides in every samples pool was then deduced from the relative signal intensities of the corresponding reporter ions. Protein identification was achieved by comparison of the peptide sequences with Specific SwissProt^®^ reviewed non-redundant database (http://www.uniprot.org/uniprot/) of *Rattus norvegicus* (rat) proteome using the Sequest^®^ HT search engine (Washington DC, U.S.A.). Abbreviations: b-ion, Precursor ion; C, Carbon; Da, Dalton; MS, Mass spectrometry; *m/z*, Mass-to-charge ratio; O, Oxygen; N, Azote; y-ion, Internal ion; U.S.A., United States of America.

**Fig 2:**
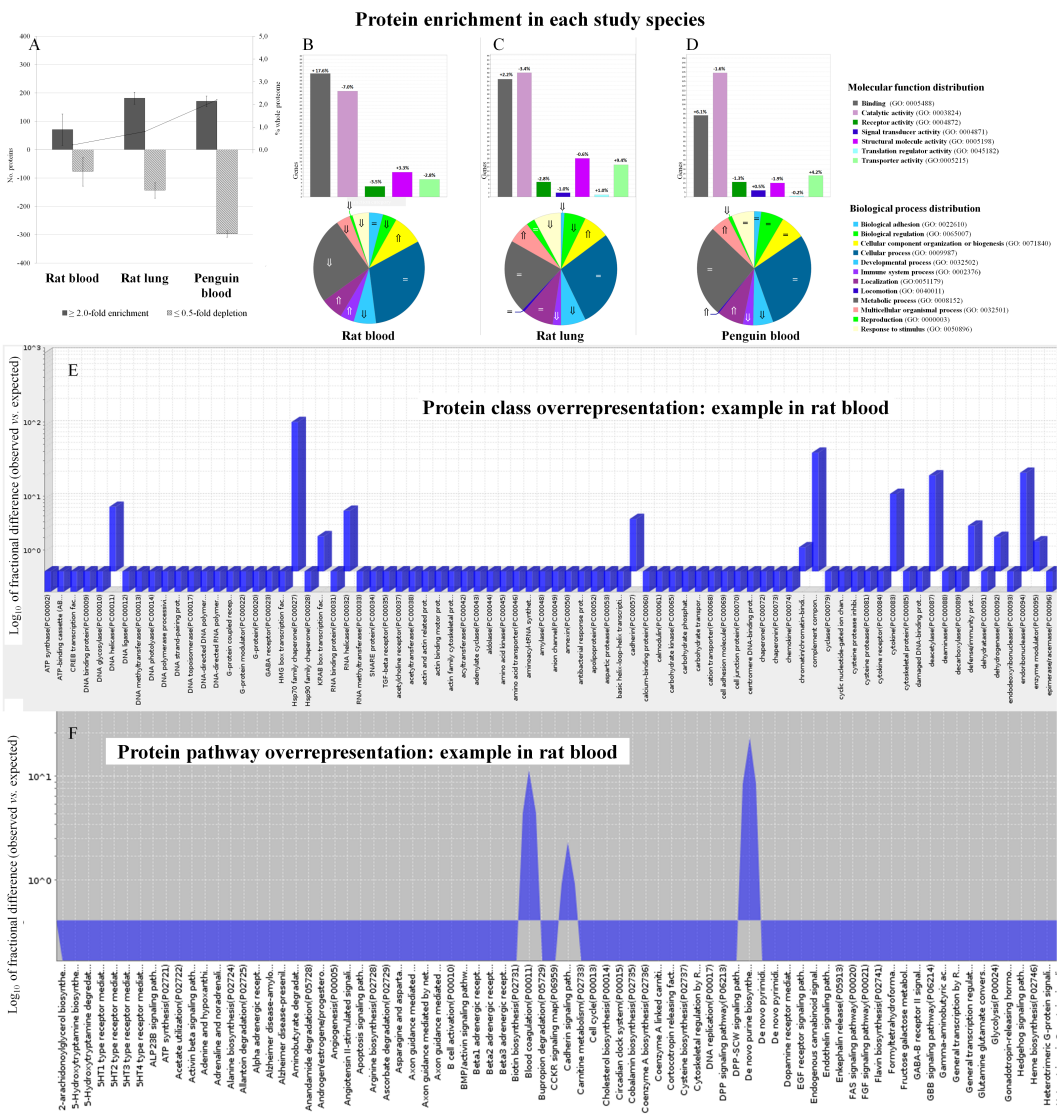
Global protein changes and Gene Ontology (GO, http://www.geneontology.org/) enrichment analysis within *Aspergillus*-diseased organisms *versus* control conditions, according to the PANTHER classification system [30,57]. A—The bar chart indicates the absolute number of proteins that were found positively or negatively ≥ 2.0-fold differentially-expressed amongst each species (S1 Table). The dots of the curve indicate the proportion of proteins, expressed in percentages in comparison to the whole known proteome of each studied organism, that were positively or negatively ≥ 2.0-fold differentially-expressed during aspergillosis; B–The multiple pie chart in the upper panel displays distribution of the molecular functions of all the proteins that were identified as positively or negatively ≥ 2.0-fold differentially-expressed in rat blood during experimental aspergillosis *vs*. control rats challenged with bacterial lipopolysaccharide (LPS); the pie chart of the lower panel shows the corresponding biological processes for the same proteins; C—Results for proteins that were positively or negatively ≥ 2.0-fold changed in rat lung during experimental aspergillosis *vs*. control rats challenged with LPS; D—Results for proteins that were positively or negatively ≥ 2.0-fold differentially represented in penguin blood during aspergillosis *vs*. control penguins undergoing non-*Aspergillus* inflammatory diseases; E–The overlaid area chart of difference obtained by bioinformatic analyses revealed that blood protein enrichment during experimental aspergillosis in rats concerned 28 distinct protein classes, including defense proteins like immunoglobulin superfamily and complement components, nucleases, various mRNA processing factors, helicases, cytokines, serine protease inhibitors and protein phosphatases/kinases; F–The bar chart of difference obtained by bioinformatic analyses revealed that blood protein enrichment during experimental aspergillosis in rats primarily concerned six different pathways: blood coagulation, cadherin signaling pathway, *de novo* purine biosynthesis, pyruvate metabolism, tricarboxylic acid cycle, and Wnt signaling pathway. Abbreviation: GO, Gene ontogeny; ⇑, positive enrichment >10% gene hit against total biological process hits; ⇓, negative enrichment >10% gene hit against total biological process hits; = , positive or negative enrichment <10% gene hit against total biological process hits.

A summarizing scheme of the iTRAQ^®^ protocol carried out in the experimental rat model is shown in Fig 1 (lung parenchyma was not sampled at baseline to avoid sacrificing healthy animals before intra-tracheal challenge) (Fig 1). Globally, the technical principle was the same in penguins. Overall, large-scale MS analysis achieved identification of 7,858 proteins in rat blood samples, 4,430 in rat lungs, and 6,436 in penguin blood specimens. Significant quantitative changes concerned a protein moiety equal to 0.6%, 1.4% and 3.0% species proteome in rat blood, rat lung and penguin blood, respectively (Fig 2A; S1 and S2 Tables).

#### Protein changes in the experimental rat model

**Fig 3:**
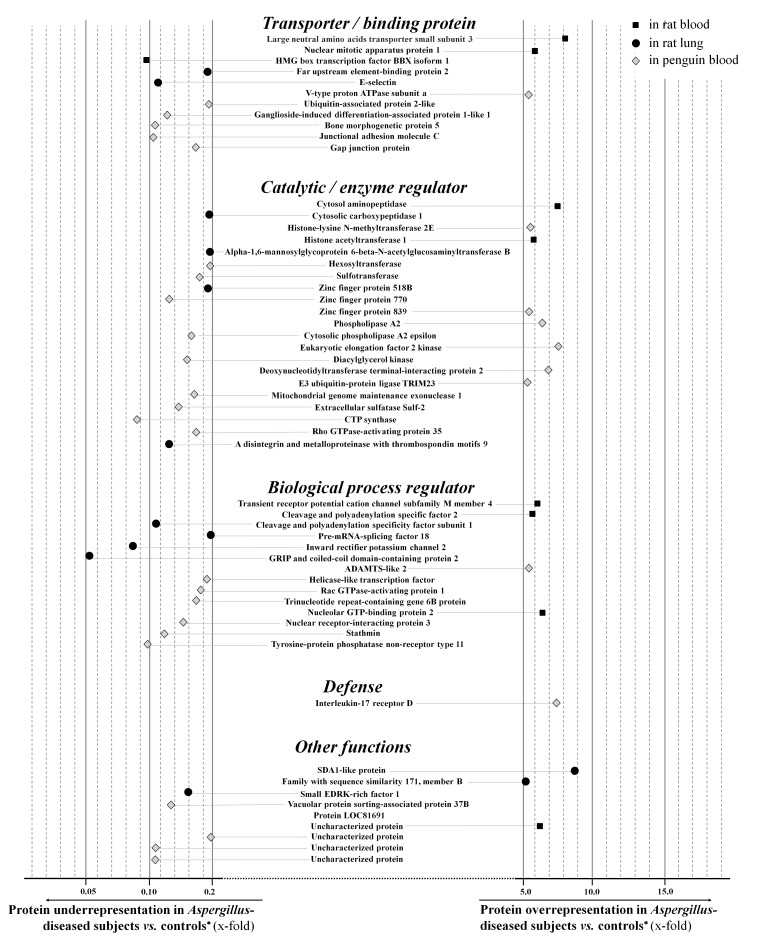
Forest plot diagram showing respective levels for the proteins that were found to be significantly overrepresented or under-represented in each animal species, in comparison with control conditions. For better clarity, only the very abundant (≥5.0-fold increased *vs*. controls) or very depleted proteins (≥5.0-fold decreased) are shown. Results are presented gathered according to protein functions and protein (sub-)families. * control conditions for rats: experimental challenge with bacterial lipopolysaccharide (LPS); for penguins: all the inflammatory non-*Aspergillus* diseases.

**Fig 4:**
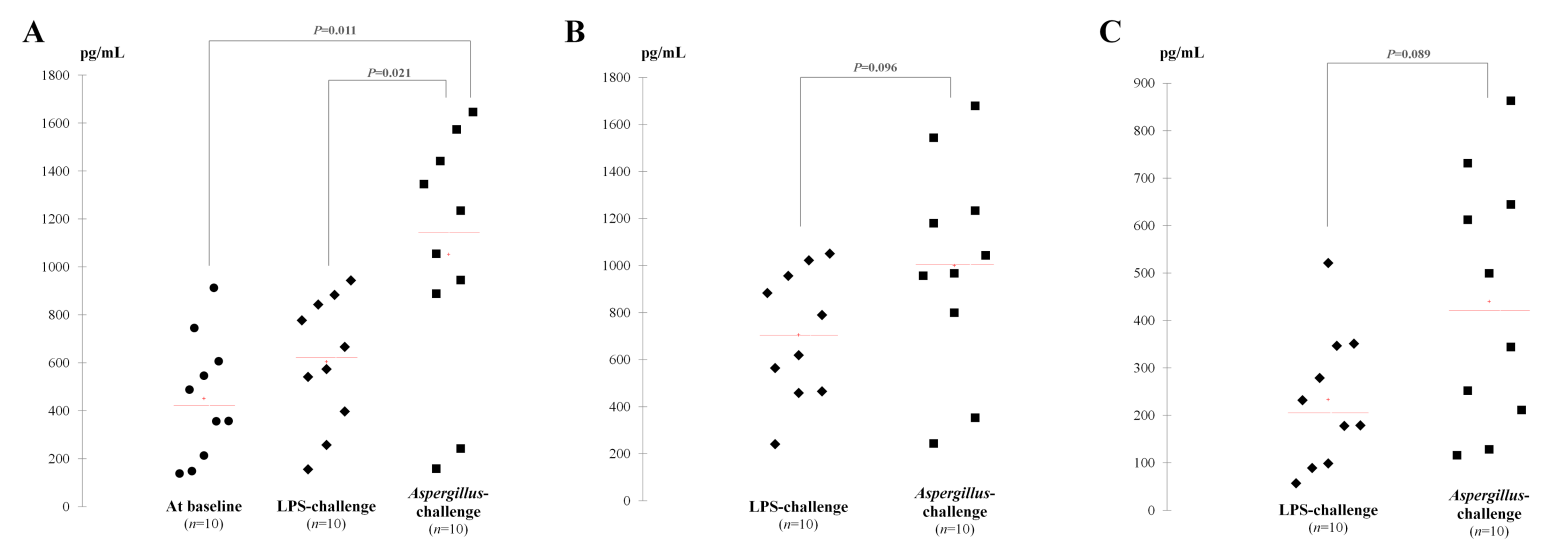
Scattergrams showing distribution of the individual measurements of Wnt1 inducible signaling pathway protein 1 (Wisp-1) and basic fibroblast growing factor (bFGF) proteins in blood or in lung from the rat experimental model. A–Wisp-1 measurements in blood from 10 rats of each group. According to the kit instructions, the detection ranged from 100 to 2,500pg/mL; B–Wisp-1 measurements in supernatants of the lung homogenates from 10 rats of each group; C–Basic FGF measurements in supernatants of the lung homogenates from 10 rats of each group. The concentration gradients of the kit standards render a theoretical kit detection range of 2–500 pg/mL through a standard curve using a vial of rat bFGF at 500 pg/mL as initial concentration. Abbreviation: LPS, bacterial lipopolysaccharide.

In rat blood, 148 proteins were ≥ 2.0-fold differentially-represented (negatively or positively) after *Aspergillus*-inoculation *vs*. LPS-challenge (Fig 2A; S1 Table). They mostly corresponded to proteins with binding function (43.8%) and catalytic activity (37.5%) and were involved in cellular process (30.9%) (Fig 2B). Among these 148 proteins, 71 were overrepresented. Ten were massively superabundant, *i*.*e*. ≥ 4.0-fold increased (Table 2, Fig 3), and were confirmed for seven by comparison with blood samples collected in healthy rats at baseline, but only moderately for the nucleolar GTP-binding protein 2, the cytosol aminopeptidase, and the anillin actin-binding protein (3.9-, 1.4-, and 2.4-fold increase) (Table 2). In comparison with rat whole proteome, 28 different protein classes appeared significantly enriched during experimental aspergillosis, including defense proteins, regulators of proteases, nucleases / helicases, phosphatases / kinases, hydrolases and deacetylases (Fig 2E). Bioinformatic analyses indicated that they were primarily involved in signaling pathways *via* cadherin and Wnt (wingless integration site protein), lipid and nucleobase metabolism, and in coagulation (Fig 2F, Table 3). Based on ELISA assay, Wnt1 inducible signaling pathway protein 1 (Wisp1) was confirmed globally overrepresented in blood of 10 *Aspergillus*-diseased rats *vs*. control rats at baseline or after LPS-challenge, 1,052.4 ± 257.6 pg/mL *vs*. 450.7 ± 128.9 pg/mL or 609.7 ± 134.6 pg/mL, respectively (*P*<0.05) (Fig 4A). Five proteins were more than 4.0-fold decreased *vs*. LPS-challenged controls, and two of them were also significantly lowered *vs*. healthy rats: long-chain-fatty-acid-CoA ligase 1 and HMG box transcription factor BBX isoform 1 (Fig 3). Notably, the major protein changes aforementioned were confirmed in a subset composed of eight distinct rat blood samples (from four LPS-challenged controls *vs*. four *Aspergillus*-challenged cases) that were individually treated by iTRAQ^®^ protocol and compared to each other (data not shown).

**Table 2:** List of the host proteins which were shown to have highly-significant increased or decreased expression (≥ 4.0-fold) in the blood of rats inoculated with *Aspergillus* conidia *versus* control rats challenged with bacterial lipopolysaccharide (LPS) or *versus* healthy rats (sampled at baseline). Fold changes in the *Aspergillus*-diseased rats (*i*.*e*. encompassing all the subjects for which blood samples were multiplexed into one single aliquot and tagged with 118-iTRAQ^®^ tag) were calculated in ratio with respect to the protein expression in healthy rats sampled at baseline (116-tag reagent) or in non-*Aspergillus*-diseased control rats undergoing inflammation due to LPS-challenge (117-tag).

Global change	Protein identification	Access. number (UniProt^®^)	Abundance ratio (fold change)		% Seq. coverage	No. unique peptide(s)	No. aa	Theoretical MW (kDa)	Calc. pI	Gene name (GenBank^®^)	Known function	Subcellular location
			*vs*. LPS controls	*vs*. baseline								
**⇑**	Large neutral amino acids transporter small subunit 3	G5BLJ1	8.052		2.074	1	482	51.8	9.96	GW7_13226		Membrane
**⇑**	Cytosol aminopeptidase	G5ANJ7	7.668	1.361	2.564	1	351	37.7	7.03	GW7_04166	metabolic process (catalytic activity; metal ion binding)	cytoplasm
**⇑**	Nucleolar GTP-binding protein 2	G5C4Q1	6.469	3.891	1.438	1	417	47.3	6.9	GW7_16109	nucleotide binding	nucleolus
**⇑**	Uncharacterized protein	D3ZNL9	6.456	5.095	5.645	1	124	13.8	10.3			
**⇑**	Transient receptor potential cation channel subfamily M member 4	G5BAF3	6.052	4.892	2.000	3	1250	138.8	7.94	GW7_13611	regulation of biological process; transport	membrane
**⇑**	Nuclear mitotic apparatus protein 1	F7FF45	5.855	8.657	1.195	2	2091	234.8	5.73	Numa1	cell differentiation; cell organization and biogenesis (protein binding)	cytoplasm; membrane; nucleus
**⇑**	Histone acetyltransferase 1	G5B6D5	5.8		3.989	1	376	41.9	7.97	GW7_11588	cell organization and biogenesis; metabolic process (catalytic activity); regulation of biological process	nucleus
**⇑**	Cleavage and polyadenylation specific factor 2 (Predicted)	D3Z9E6	5.732	5.468	1.023	1	782	88.3	5.11	Cpsf2	metabolic process	membrane
**⇑**	Uncharacterized protein	M0RCJ6	4.874		6.122	1	147	16.5	4.23		metal ion binding	
**⇑**	Anillin, actin-binding protein	D4A026	4.026	2.444	3.297	2	1122	122.7	7.56	Olr519	response to stimulus	membrane
**⇓**	Myosin-XVIIIb	G5BGN6	0.248	0.187	1.458	2	2536	282.1	6.9	GW7_20837	cell organization and biogenesis (catalytic activity); metabolic process (motor activity;); regulation of biological process (nucleotide binding; protein binding)	cytoplasm; cytoskeleton
**⇓**	Uncharacterized protein C3orf67	A0A091D425	0.242	0.294	3.790	1	686	76.6	6.02	H920_13424	regulation of biological process (nucleotide binding)	nucleus
**⇓**	Olfactory receptor	A0A091DMC7	0.231	0.214	2.866	1	314	35	9.14	LOC104847747	regulation of biological process; response to stimulus (receptor activity; signal transducer activity)	membrane
**⇓**	Long-chain-fatty-acid—CoA ligase 1	P18163	0.209	0.57	3.004	1	699	78.1	6.99	Acsl1	metabolic process (catalytic activity); regulation of biological process; response to stimulus; transport (nucleotide binding)	endoplasmic reticulum; membrane; mitochondrion
**⇓**	HMG box transcription factor BBX isoform 1	A0A0P6JTL2	0.121	0.804	0.851	1	940	104.8	8.59	BBX	DNA binding	nucleus

Abbreviations: aa, Amino acids; Access., Accession; calc., Calculated; Exp., Expected; kDa, Kilo Dalton; LPS, Bacterial lipopolysaccharide; MW, Molecular weight; No, Number; pI, Isoelectric point; Seq., Sequence

**Table 3:** List of the PANTHER pathways that were enriched during aspergillosis *versus* control situations. For each species, enrichment was calculated in ratio with respect to the pathway representations reported in the reference lists (http://www.geneontology.org/, as mentioned in the *Material & Methods*). Are shown below in the table only the PANTHER pathways that were significantly enriched for at least one of the species studied (*P*<0.05).

	Rat*		Penguin^$^
	blood	lung	blood
**5HT1 type receptor mediated signaling pathway**			9.6
**5HT2 type receptor mediated signaling pathway**			4.0
**5HT4 type receptor mediated signaling pathway**			4.4
**Alzheimer disease-amyloid secretase pathway**			2.0
**Alzheimer disease-presenilin pathway**			1.1
**Angiogenesis**		2.2	
**Apoptosis signaling pathway**			1.3
**Axon guidance mediated by netrin**			3.6
**Axon guidance mediated by Slit/Robo**			11.5
**B-cell activation**			1.8
**Beta1 adrenergic receptor signaling pathway**			3.4
**Beta2 adrenergic receptor signaling pathway**			3.5
**Beta3 adrenergic receptor signaling pathway**			5.5
**Blood coagulation**	11.9	4.1	
**Cadherin signaling pathway**		1.4	1.1
**CCKR signaling map**		1.2	
**Cholesterol biosynthesis**		11.5	
**Cytoskeletal regulation by Rho GTPase**		2.6	
***De novo* purine biosynthesis**	19.1		
**DNA replication**			13.8
**Dopamine receptor mediated signaling pathway**			2.5
**EGF receptor signaling pathway**		1.4	
**Endothelin signaling pathway**			2.7
**Enkephalin release**			4.3
**FGF signaling pathway**		2.0	1.1
**Fructose galactose metabolism**			25.6
**GABA-B receptor II signaling**			4.1
**General transcription by RNA polymerase I**			8.2
**Glycolysis**		3.9	14.4
**Hedgehog signaling pathway**		10.3	
**Heme biosynthesis**			12.8
**Heterotrimeric G-protein signaling pathway-Gi alpha and Gs alpha mediated pathway**			3.9
**Heterotrimeric G-protein signaling pathway-Gq alpha and Go alpha mediated pathway**			2.1
**Heterotrimeric G-protein signaling pathway-rod outer segment phototransduction**		5.8	
**Histamine H1 receptor mediated signaling pathway**			2.8
**Histamine H2 receptor mediated signaling pathway**			6.4
**Huntington disease**		1.2	
**Inflammation mediated by chemokine and cytokine signaling pathway**			1.7
**Insulin/IGF pathway-mitogen activated protein kinase kinase/MAP kinase cascade**		5.8	
**Integrin signalling pathway**			1.4
**Interleukin signaling pathway**		2.1	
**Ionotropic glutamate receptor pathway**			2.6
**Lipoate_biosynthesis**			105.1
**Lysine biosynthesis**		98.1	
**Metabotropic glutamate receptor group I pathway**			11.5
**Metabotropic glutamate receptor group III pathway**			1.9
**Muscarinic acetylcholine receptor 1 and 3 signaling pathway**			4.7
**Nicotine pharmacodynamics pathway**			3.5
**Nicotinic acetylcholine receptor signaling pathway**			
**O-antigen biosynthesis**			23.0
**Opioid prodynorphin pathway**			4.4
**Opioid proenkephalin pathway**			4.3
**Oxidative stress response**			2.3
**Oxytocin receptor mediated signaling pathway**			2.3
**p38 MAPK pathway**			3.1
**p53 pathway**			1.6
**Parkinson disease**			
**PDGF signaling pathway**		2.7	
**Pentose phosphate pathway**			14.4
**PI3 kinase pathway**			
**Pyruvate metabolism**	47.8	16.4	
**Ras pathway**		2.6	
**T-cell activation**			
**Tricarboxylic acid cycle**	52.1		
**TGF-beta signaling pathway**		2.0	2.8
**Thyrotropin-releasing hormone receptor signaling pathway**			2.3
**Ubiquitin proteasome pathway**			6.4
**VEGF signaling pathway**			1.9
**Wnt signaling pathway**	3.9	1.3	1.9

* *vs*. bacterial lipopolysaccharides (LPS)-challenged control rats

^**$**^ *vs*. non-*Aspergillus* inflammatory control penguins

In rat lungs infected with *Aspergillus*, 325 proteins were differentially over- or under-represented *vs*. LPS inflammatory controls (Fig 2A; S1 Table). Among them, 37.2% and 35.3% had catalytic and binding activities, whereas 28.1% and 21.2% were in charge of cellular and metabolic processes (Fig 2C), respectively. One hundred eighty-two proteins were at least 2.0-fold increased, but only 14 were more than 4.0-fold increased (Fig 3; Table 4). Notably, although none of them was also found overrepresented in rat blood in the same time, similar or overlapping pathways were promoted in both sample types: for example, lipid metabolism *via* cholesterol and pyruvate biosynthesis, coagulation, cadherin and Wnt signaling pathways (Table 3). As a confirmation, ELISA assay showed individual levels for Wnt1 inducible signaling pathway protein 1 (Wisp-1) that were insignificantly higher in lung homogenates from *Aspergillus*-diseased rats than in LPS-challenged rats, 999.5 ± 228.6 pg/mL *vs*. 705.1 ± 137.8 pg/mL (*P*>0.05) (Fig 4B). Likewise, the fibroblast growing factor (FGF) pathway was found 2.0-fold enriched in *Aspergillus*-diseased rats *vs*. those challenged with LPS, and this trend was confirmed through the ELISA assay, 440.0 ± 133.1 pg/mL *vs*. 233.2 ± 122.2 pg/mL (*P*>0.05) (Fig 4C). Overall, relative enrichment applied to 55 distinct protein classes: transporters, nucleic acid binding, ion-channel, serine protease inhibitor, and helicase families were primarily represented in rat lungs. In addition, 143 proteins were found at least 2.0-fold negatively changed *vs*. LPS-inflammatory rat lungs. Interestingly, D-amino-acid oxidase protein, which was 4.9-fold decreased in lungs of *Aspergillus*-challenged rats, was also significantly lowered in blood by 2.1-fold (Tables 3 and 4). Likewise, olfactory receptor and coiled-coil domain-containing protein were found moderately low in *Aspergillus*-diseased rat lungs *vs*. LPS-challenged control lungs, reduced by 2.4- and 2.7-fold, respectively, and also in blood, 4.7- and 2.9-fold, respectively.

**Table 4:** List of the host proteins which were shown to have highly-significant increased or decreased expression (≥ 4.0-fold) in the lung parenchyma of rats inoculated with *Aspergillus* conidia *versus* control rats challenged with bacterial lipopolysaccharide (LPS). Fold changes in the diseased rats (*i*.*e*. encompassing all the subjects for which blood samples were multiplexed into one single aliquot and tagged with 121-iTRAQ^®^ tag) were calculated in ratio with respect to the protein expression in non-*Aspergillus*-diseased control rats undergoing inflammation due to LPS-challenge (119-tag reagent).

Global changes	Protein identification	Access. number (UniProt^®^)	Abundance ratio (fold change *vs*. LPS-challenged controls)	% Seq. coverage	No. unique peptide(s)	No. aa	Theoretical MW (kDa)	Calc. pI	Gene name (GenBank^®^)	Known function	Subcellular location
**⇑**	SDA1-like protein	G5ARD1	8.927	1.902173913	2	368	42.5	9.42	GW7_16137	cell organization and biogenesis; transport	
**⇑**	Family with sequence similarity 171, member B	D3ZTG3	5.011	1.973684211	1	760	84.8	7.9	Fam171b		membrane
**⇑**	Uncharacterized protein DUF4706	G5BQI7	4.778	12.34567901	2	243	27.3	5.16	GW7_00817		
**⇑**	Apoptosis-stimulating of p53 protein 1	A0A091DHP4	4.649	1.705029838	1	1173	128.3	6.62	Ppp1r13b	cell death; regulation of biological process (protein binding)	
**⇑**	Poly(RC)-binding protein 2	A0A091CSJ9	4.52	5.995717345	1	467	48.7	7.64	Pcbp2	RNA binding	
**⇑**	Homeobox C13	D3ZKV8	4.504	7.317073171	2	328	35.2	8.95	Hoxc13	metabolic process; regulation of biological process (DNA binding)	nucleus
**⇑**	Mitochondrial fission regulator 1	G3V9A7	4.438	2.388059701	1	335	36.8	9.2	Mtfr1	cell organization and biogenesis; metabolic process	cytoplasm; membrane; mitochondrion
**⇑**	DEP domain-containing protein 1A	A0A091CZB3	4.434	1.303317536	1	844	95.9	8.51	Depdc1	regulation of biological process; response to stimulus	
**⇑**	Histone-lysine N-methyltransferase MLL4 (Fragment)	G5BXN0	4.411	1.273148148	2	2592	281.1	7.93	GW7_18413	metabolic process (catalytic activity; DNA binding; metal ion binding; protein binding)	nucleus
**⇑**	Putative sodium-coupled neutral amino acid transporter 11	D4A335	4.358	6.962025316	1	158	17.2	7.5	Slc38a11		membrane
	Centrobin	A0A091CMZ6	4.31	5.066079295	2	908	102.2	5.67	Cntrob		
**⇑**	Remodeling and spacing factor 1	D3ZGQ8	4.218	1.078582435	2	1298	146.3	5.3	Rsf1	cell organization and biogenesis; metabolic process; regulation of biological process (metal ion binding; protein binding)	nucleus
**⇑**	Pyruvate kinase	A0A091D3V4	4.184	3.482587065	2	603	64.9	8.88	Pklr	metabolic process (catalytic activity; metal ion binding)	
**⇑**	SET-binding protein 1	F1M4Q7	4.107	3.552123552	3	1295	141.5	9.89	Setbp1	DNA binding	nucleus
**⇓**	BRCA2 and CDKN1A-interacting protein	D3ZB65	0.249	3.243243243	1	370	41.7	4.67	Bccip	cell differentiation; regulation of biological process (enzyme regulator activity; RNA binding); response to stimulus	cytoplasm
**⇓**	Trichohyalin	G5B8K2	0.243	1.440329218	1	486	61.1	6.23	GW7_10296	metal ion binding	
**⇓**	Uncharacterized protein DUF4573 (Fragment)	G5B8Y3	0.242	1.759530792	1	341	35.8	4.42	GW7_21032		
**⇓**	Inosine-5'-monophosphate dehydrogenase 1	A0A091DB15	0.235	1.144492132	1	699	75.2	7.66	Impdh1	metabolic process (catalytic activity; metal ion binding; nucleotide binding)	cytoplasm; nucleus
**⇓**	MDS1 and EVI1 complex locus protein EVI1 isoform b	A0A0P6JA19	0.234	3.631961259	2	1239	139.4	6.47	Mecom	regulation of biological process (DNA binding; metal ion binding; protein binding)	
**⇓**	Tripartite motif protein 36 (Predicted)	F1LY63	0.223	3.566529492	2	729	82.8	6.32	Trim36	metabolic process (catalytic activity); regulation of biological process (metal ion binding; protein binding); transport	extracellular exosome
**⇓**	Uncharacterized protein	D3ZNL9	0.221	5.64516129	1	124	13.8	10.3			
**⇓**	D-amino-acid oxidase	A0A091CVS3	0.204	3.738317757	1	428	47.8	7.96	Dao	metabolic process (metal ion binding)	membrane
**⇓**	Pre-mRNA-splicing factor 18	A0A091DSA9	0.197	1.41955836	1	634	70.9	9.07	Prpf18	metabolic process	spliceosomal complex
**⇓**	Alpha-1,6-mannosylglycoprotein 6-beta-N-acetylglucosaminyltransferase B	A0A091EJH1	0.191	0.663716814	1	904	101	8.09	Mgat5b	metabolic process (catalytic activity)	membrane
**⇓**	Cytosolic carboxypeptidase 1	A0A091CYT4	0.189	1.206757844	1	1243	139.4	6.37	Agtpbp1	cell differentiation; metabolic process (catalytic activity; metal ion binding)	
**⇓**	Far upstream element-binding protein 2	A0A091DEW1	0.187	0.712589074	1	842	89.5	8.73	H920_09737	Transport (RNA binding)	membrane
**⇓**	Zinc finger protein 518B	A0A091D5L9	0.185	4.396984925	2	796	88.1	9.29	Znf518b	metabolic process; regulation of biological process (metal ion binding)	nucleus
**⇓**	Small EDRK-rich factor 1	G5BSZ4	0.159	8.43373494	1	83	9.6	10.29	LOC101723724		
**⇓**	A disintegrin and metalloproteinase with thrombospondin motifs 9	G5B0Q1	0.128	0.545596259	1	1283	141.8	7.71	GW7_00190	metabolic process (catalytic activity); regulation of biological process (metal ion binding); response to stimulus	proteinaceous extracellular matrix
**⇓**	E-selectin	A0A091DFV2	0.117	3.671706263	2	926	103.3	6.38	H920_09505	protein binding	membrane
**⇓**	Cleavage and polyadenylation specificity factor subunit 1	A0A091DK29	0.113	0.416377516	1	1441	160.8	6.35	Cpsf1		nucleus
**⇓**	Inward rectifier potassium channel 2	A0A091DU44	0.084	1.517067004	1	791	87.1	8.24	Kcnj2	regulation of biological process; transport	membrane
**⇓**	GRIP and coiled-coil domain-containing protein 2	G5CB89	0.046	0.982091277	2	1731	200.6	5.17	GW7_05151	metabolic process; transport (protein binding)	

Abbreviations: aa, Amino acids; Access., Accession; calc., Calculated; Exp., Expected; kDa, Kilo dalton; LPS, Bacterial lipopolysaccharides; MW, Molecular weight; No, Number; pI, Isoelectric point; Seq., Sequence.

#### Large protein changes in penguin samples

In blood collected from penguins with aspergillosis, 468 proteins were differentially over- or under-expressed *vs*. non-*Aspergillus* inflammatory controls (Fig 2A; S1 Table). Among them, 31.7% expressed binding activities, whereas 29.1% and 26.2% were involved in cellular and metabolic processes (Fig 2D). Individually, 171 proteins were significantly overrepresented, including 17 more than 4.0-fold increased, and 135 of them were confirmed *vs*. clinically-normal controls (Fig 3; Table 5). Protein enrichment applied to 63 families, including various transfer / carrier, receptor, nucleic acid binding, nuclease, metalloprotease, and serine protease inhibitor proteins. Receptor and apoptosis pathways, as well as signaling pathways *via* cadherin and Wnt, were overrepresented during aspergillosis in comparison with physiological pathways of the whole proteome (Table 3). While they were 4.4-, 2.5-, 2.5-, 2.2-fold overexpressed *vs*. non-*Aspergillus* inflammatory controls, F-box/LRR-repeat protein 4, THAP domain-containing protein 1, histidine-tRNA cytoplasmic ligase and AIM1 (absent in melanoma-1) protein were found also statistically overrepresented *vs*. convalescent penguins, 2.5-, 2.1-, 2.0- and 3.8-fold, respectively. Conversely, 297 proteins were found decreased in *Aspergillus*-diseased penguins in comparison with non-*Aspergillus* inflammatory controls. The decrease was confirmed *vs*. clinically-normal controls for 30 of the 39 proteins lowered by ≥4.0-fold, and 13 were also found lowered *vs*. convalescent penguins. These major protein changes were confirmed in eight distinct penguin blood samples that were individually treated by iTRAQ^®^ protocol and compared to each other (data not shown), *e*.*g*. the eukaryotic elongation factor 2 kinase was found 2.0-, 2.1-, 2.8- and 2.6-fold overrepresented in the four diseased cases *vs*. the four healthy controls, while the fragment of interleukin-17 receptor D was 5.4-, 7.6-, 6.3-, and 6.8- fold increased.

**Table 5:** List of the host proteins which were shown to have highly-significant increased or decreased expression (≥ 4.0-fold) in penguin blood during *Aspergillus*-disease *versus* non-*Aspergillus* inflammatory controls or *versus* healthy controls or *versus* convalescent penguins. Fold changes in the diseased penguins (*i*.*e*. encompassing all the subjects for which blood samples were multiplexed within one single aliquot and tagged with 115-iTRAQ^®^ tag) were calculated in ratio with respect to the protein expression in non-*Aspergillus* diseased control penguins undergoing inflammation due to any miscellaneous cause (114-tag reagent), or in clinically-normal penguins (113-tag) and in convalescent birds (116-tag).

	Protein identification	Access. number (UniProt^®^)	Abundance ratio (fold change)			% Seq. coverage	No. unique peptide(s)	No. aa	Theoretical MW (kDa)	Calc. pI	Gene name (GenBank^®^)	Known function	Subcellular location
Global changes			*vs*. inflammatory controls	*vs*. clinically-normal controls	*vs*. convalescents								
**⇑**	Eukaryotic elongation factor 2 kinase	A0A087R6I1	7.629	3.22	0.384	4.275	2	725	82.7	5.34	EEF2K	metabolic process (catalytic activity; metal ion binding; nucleotide binding; protein binding; RNA binding)	
**⇑**	Interleukin-17 receptor D (Fragment)	A0A093NMA6	7.58	8.498	0.436	4.154	3	698	79.2	6.79	IL17RD	regulation of biological process (protein binding); response to stimulus	
**⇑**	Deoxynucleotidyltransferase terminal-interacting protein 2 (Fragment)	A0A087QMM8	6.996	7.263	0.304	4.189	3	716	80.1	5.49	DNTTIP2	metabolic process (catalytic activity)	
**⇑**	Phospholipase A2 (Fragment)	A0A087QKZ2	6.461	6.371	0.695	16.279	1	129	14.5	8.62	PLA2G2E	metabolic process (catalytic activity); transport (metal ion binding)	extracellular
**⇑**	Histone-lysine N-methyltransferase 2E	A0A087RKR4	5.667	6.674	0.535	1.063	1	1787	197.1	7.39	KMT2E	metabolic process (catalytic activity; metal ion binding; protein binding)	
**⇑**	Zinc finger protein 839 (Fragment)	A0A087R591	5.505	2.798	0.797	6.586	1	167	18.2	4.54	ZNF839	transport (metal ion binding)	
**⇑**	V-type proton ATPase subunit a	A0A087RJJ5	5.456	6.377	0.252	4.869	2	842	96.1	6.47	ATP6V0A4	transport	membrane
**⇑**	ADAMTS-like 2 (Fragment)	A0A087RH31	5.334	100	0.619	3.444	2	871	97.9	7.77	ADAMTSL2	metabolic process (catalytic activity; metal ion binding)	proteinaceous extracellular matrix
**⇑**	E3 ubiquitin-protein ligase TRIM23 (Fragment)	A0A087QP33	5.278	7.026	0.361	8.592	3	547	61.1	6.27	TRIM23	regulation of biological process (catalytic activity; metal ion binding; nucleotide binding; protein binding;); response to stimulus (signal transducer activity)	
**⇑**	DNA replication ATP-dependent helicase/nuclease DNA2 (Fragment)	A0A087QMS4	4.921	6.753	0.365	5.728	4	995	112.2	8.35	DNA2	cell organization and biogenesis; metabolic process (catalytic activity);transport (nucleotide binding)	
**⇑**	Epsin-3 (Fragment)	A0A093NY33	4.682	1.815	0.402	1.616	1	433	47.7	5.25	EPN3		
**⇑**	BMP and activin membrane-bound inhibitor (Fragment)	A0A087QU69	4.542	6.657	0.329	14.830	1	236	26.3	7.64	BAMBI	regulation of biological process	membrane
**⇑**	Anoctamin (Fragment)	A0A093NFJ6	4.4	3.352	0.39	6.433	3	715	83.5	8.48	ANO9	cell organization and biogenesis (protein binding)	membrane
**⇑**	F-box/LRR-repeat protein 4	A0A087QUK7	4.355	0.595	2.451	3.870	2	620	70.5	6.39	FBXL4	protein binding	
**⇑**	Uncharacterized protein (Fragment)	A0A087QID6	4.314	4.919	0.232	15.217	1	92	10.4	5.4	AS27_07119	defense response; metabolic process (catalytic activity);response to stimulus	
**⇑**	Uncharacterized protein (Fragment)	A0A093RKS1	4.272	1.479	0.231	15.151	1	66	7.9	11.02			
**⇑**	Serine/threonine-protein kinase TAO1	A0A093NM90	4.140	1.307	0.993	3.796	3	1001	115.9	7.65	TAOK1	metabolic process (catalytic activity; nucleotide binding)	
**⇓**	6-phosphogluconate dehydrogenase, decarboxylating (Fragment)	A0A087QL53	0.249	0.137	3.324	10.995	3	482	53.2	7.25	PGD	metabolic process (catalytic activity)	
**⇓**	Ribosomal protein L1 (Fragment)	A0A093NMM0	0.244	0.24	4.784	16.402	1	189	21.6	9.57	LOC103926424		
**⇓**	Nicotinic acetylcholine receptor (Fragment)	A0A087QRY0	0.241	1.019	0.798	9.871	2	466	53.4	7.43	CHRNB4	transport	membrane
**⇓**	Latrophilin-3 (Fragment)	A0A087QR75	0.24	0.41	1.924	5.468	4	1463	162.9	6.57	LPHN3	regulation of biological process (receptor activity);response to stimulus (transducer activity)	membrane
**⇓**	Trinucleotide repeat-containing gene 6C protein (Fragment)	A0A087R2H6	0.24	0.36	1.967	2.716	2	1730	180.7	6.2	TNRC6C	regulation of biological process (nucleotide binding; protein binding)	
**⇓**	Regulation of nuclear pre-mRNA domain-containing protein 2 (Fragment)	A0A087QP85	0.236	0.731	0.777	1.187	1	1179	125.6	6.46	RPRD2		
**⇓**	F-box only protein 8	A0A087QNV4	0.232	0.338	2.965	4.702	2	319	36.9	8.12	FBXO8	regulation of biological process (protein binding)	
**⇓**	Parafibromin (Fragment)	A0A087RDT0	0.227	0.087	3.834	9.445	2	487	55.5	9.64	AS27_00346	cell organization and biogenesis; metabolic process	Cdc73/Paf1 complex
**⇓**	Uncharacterized protein CXorf57 (Fragment)	A0A087QUG5	0.223	0.367	3.291	7.020	3	698	79.8	6.62	LOC103895704		
**⇓**	Sodium channel protein	A0A093P706	0.223	0.289	2.989	3.128	4	2014	228.9	5.55	SCN2A	regulation of biological process (protein binding);transport	membrane
**⇓**	Katanin p60 ATPase-containing subunit A-like 1	A0A087QM85	0.219	0.294	2.552	6.339	2	489	55.1	6.74	KATNAL1	metabolic process (catalytic activity; nucleotide binding; protein binding); response to stimulus	cytoplasm
**⇓**	Procollagen galactosyltransferase 2 (Fragment)	A0A087QVF2	0.219	0.214	2.115	8.623	2	545	63.6	6.49	COLGALT2	metabolic process (catalytic activity)	
**⇓**	Anaphase-promoting complex subunit 2 (Fragment)	A0A087RAE7	0.219	0.164	3.805	6.641	3	783	90.1	5.05	ANAPC2	metabolic process (protein binding)	
**⇓**	Tyrosine-protein kinase receptor (Fragment)	A0A087RB90	0.216	0.695	0.943	0.973	1	1336	151.1	6.13	INSR	metabolic process (catalytic activity);regulation of biological process (nucleotide binding; protein binding; receptor activity);response to stimulus (signal transducer activity0	membrane
**⇓**	Ras-related protein Rab-44 (Fragment)	A0A093NRM9	0.216	0.176	4.296	8.571	2	210	23.7	7.42	RAB44	regulation of biological process (nucleotide binding); response to stimulus	
**⇓**	Uncharacterized protein (Fragment)	A0A093NPJ6	0.197	0.27	2.787	4.761	1	168	18.1	8.92	DDIAS		
**⇓**	Hexosyltransferase (Fragment)	A0A093PG72	0.195	0.116	4.597	4.356	1	505	58.2	5.47	CSGALNACT2	catalytic activity	membrane
**⇓**	Ubiquitin-associated protein 2-like	A0A087R9Y4	0.193	0.201	2.343	2.626	2	1104	116.2	7.2	UBAP2L	protein binding	
**⇓**	Helicase-like transcription factor (Fragment)	A0A087RBS0	0.185	0.167	0.982	2.808	3	997	111.7	8.22	HLTF	metabolic process (catalytic activity); regulation of biological process (metal ion binding; nucleotide binding; protein binding);response to stimulus	
**⇓**	Rac GTPase-activating protein 1	A0A087QSR1	0.178	0.84	1.205	4.754	2	631	70.6	8.68	RACGAP1	regulation of biological process (metal ion binding);response to stimulus	
**⇓**	Sulfotransferase	A0A087QKL3	0.175	0.088	2.464	6.361	1	393	45.4	9.31	LOC103907276	metabolic process (catalytic activity)	membrane
**⇓**	Rho GTPase-activating protein 35 (Fragment)	A0A087R6Q4	0.171	0.135	6.634	5.164	4	1123	128.2	6.23	ARHGAP35	regulation of biological process (nucleotide binding);response to stimulus	
	Gap junction protein	A0A087RIK3	0.169	0.178	4.774	2.290	1	393	45.2	6.84	GJC1	cell communication	membrane
**⇓**	Trinucleotide repeat-containing gene 6B protein (Fragment)	A0A087QLS9	0.169	0.147	4.742	3.072	2	1595	170.2	8.13	TNRC6B	regulation of biological process (nucleotide binding)	
**⇓**	Mitochondrial genome maintenance exonuclease 1 (Fragment)	A0A087QIY3	0.166	0.955	0.587	24.411	3	340	39	8.85	MGME1	metabolic process (catalytic activity);response to stimulus	mitochondrion
**⇓**	Cytosolic phospholipase A2 epsilon (Fragment)	A0A093NHY0	0.161	0.175	2.912	7.303	2	356	41	6.86		metabolic process (catalytic activity)	
**⇓**	Diacylglycerol kinase (Fragment)	A0A087RCC7	0.157	0.093	2.935	2.376	3	1136	125.9	8.24	DGKK	metabolic process (catalytic activity;);regulation of biological process (metal ion binding; nucleotide binding; protein binding); response to stimulus	
**⇓**	Nuclear receptor-interacting protein 3 (Fragment)	A0A087RK91	0.152	0.216	6.157	7.296	1	233	25.9	8.41	NRIP3	metabolic process (catalytic activity)	
**⇓**	Extracellular sulfatase Sulf-2	A0A093P9Q8	0.143	1.509	3.025	5.479	4	876	102.1	9.16	SULF2	metabolic process (catalytic activity; metal ion binding)	cell surface; endoplasmic reticulum; Golgi
**⇓**	Vacuolar protein sorting-associated protein 37B (Fragment)	A0A087RB33	0.135	0.471	1.17	4.296	1	256	28.4	9.19	VPS37B		
**⇓**	Zinc finger protein 770 (Fragment)	A0A087QME4	0.134	0.181	4.667	2.687	2	707	82.4	9.26	ZNF770	metabolic process (metal ion binding);regulation of biological process	nucleus
**⇓**	Ganglioside-induced differentiation-associated protein 1-like 1	A0A087R7M3	0.128	0.741	0.819	1.907	1	367	42.2	6.74	GDAP1L1	protein binding	membrane
**⇓**	Stathmin (Fragment)	A0A093RH12	0.122	0.872	0.828	10.32	2	155	18.2	7.99	STMN3	cell organization and biogenesis; regulation of biological process (protein binding)	cytoplasm
**⇓**	Bone morphogenetic protein 5	A0A087R301	0.111	0.144	1.313	6.181	3	453	51.6	8.7	BMP5	protein binding	extracellular
**⇓**	Uncharacterized protein (Fragment)	A0A093RP45	0.111	0.116	56.632	3.768	2	345	38.7	8.91	AS28_04863		
**⇓**	Uncharacterized protein (Fragment)	A0A087R1N1	0.109	0.084	4.479	4.494	1	356	40.8	9.6	LOC103898708		membrane
**⇓**	Junctional adhesion molecule C (Fragment)	A0A093NNZ5	0.108	0.265	1.37	2.047	1	293	32.7	7.3	JAM3	protein binding	membrane
**⇓**	Tyrosine-protein phosphatase non-receptor type 11 (Fragment)	A0A087QSB7	0.099	0.67	0.775	34.61	1	104	11.8	7.71	LOC103894741	regulation of biological process (receptor activity)	
**⇓**	CTP synthase	A0A093NNY0	0.076	0.063	1.728	12.434	5	571	64.4	5.96	CTPS1	metabolic process (catalytic activity; nucleotide binding)	

Abbreviations: aa, Amino acids; Access., Accession; calc., Calculated; Exp., Expected; kDa, Kilo dalton; MW, Molecular weight; No, Number; pI, Isoelectric point; Seq., Sequence

## Discussion

In both human and veterinary medicine, routine diagnosis and curative treatment of aspergillosis continue to be very difficult [1]. For example, although *Aspergillus* galactomannan antigen detection has nowadays been acknowledged as an important tool in neutropenic human patients [6,31], the present work confirmed important diagnostic limitations regarding its reliability in veterinary samples, since in this study only 23.1% of the *Aspergillus*-diseased penguins had galactomannan index > 0.7. Overall, it is critical to search for new biomarkers of aspergillosis in order to improve its diagnosis, and consequently to decrease the subsequent morbidity and mortality by initiating rapid adequate antifungal therapy [32]. Pathophysiology studies can bring new insights for such a purpose, as well as for suggesting new therapeutic targets.

Through its great sensitivity, proteomics may contribute to the discovery of new markers of aspergillosis [14]. In such a context, iTRAQ^®^ protocol appears as a convincing innovative technique based on a ready-to-use affordable kit that allows a direct differential comparison of proteomes [23,33]. Noteworthy, iTRAQ^®^ method is only programmed to make relative comparison of proteins that are detectable in all the analyzed samples. It means that this technique cannot be used to detect protein(s) of pathogens that are supposed to be present only in infected fluids/tissues and not in healthy specimens. It is also the reason why iTRAQ^®^ is rather dedicated to investigate host protein changes [34]. Other molecular tools focused on pathogen cell wall, intracellular components or extracellular secretome should be considered in order to yield better rating in terms of the dynamic range of the analysis and microorganism specificity. To address host protein changes during *Aspergillus* disease, our iTRAQ^®^ comparisons were carried out in pooled samples from two distinct animal models, in order to reduce inter-individual variabilities amongst each group and to increase the number of included specimens [23]. Noteworthy, one should be aware that this approach limits how the global results can be interpreted: specifically, it is indeed not possible to identify if the result from a single data point–with aberrant value–is causing skewing of the entire dataset. However, this limitation should not be a problem for the initial phase of screening, like in this preliminary work, especially if a confirmation technique is subsequently performed for individual measurements, like trough ELISA assays, and if the animal models are quite homogenous like ours. Experimental infection yields in the present study were similar to that previously reported >90% for rats [19], although they nonetheless underscored the critical need for systematically verifying the correct disease categorization by the means of several surrogate endpoints including galactomannan antigen, fungal cultures, and histopathology [14,19]. To include relevant controls for the proteomic analysis, a comparison of *Aspergillus*-infected rats with control rats experiencing inflammation by bacterial endotoxin was performed [14], because the latter generates major inflammation that can be observed with either lung infection or inhalation of toxic particles. However, the infection induction mimics that in neutropenic human patients only, so that conclusions should not be extended to non-neutropenic conditions [35]. Regarding penguins, we rigorously enforced recruitment of miscellaneous controls which included various inflammatory processes and other non-aspergillosis infectious diseases. Nonetheless, one can notice that the mean age in the control group is a little bit higher (*P* = 0.044), so than can introduce a potential bias: this demographic discrepancy is due to the natural course of aspergillosis that is rather acute, while most other control inflammatory diseases maybe occur more chronically.

The spectrum of all the proteins that were found differentially-represented during aspergillosis in rats and penguins showed major involvement of those with binding functions. Differences of protein expression were observed between fluids and tissues, suggesting distinct metabolic patterns depending on the anatomic site of infection [36]. Additionally iTRAQ^®^ analysis underlined great expression differences, both qualitative and quantitative, between the studied species: as illustrated previously in birds [37], penguins showed a lot of protein variations during aspergillosis, more than what was observed in our rat model. A possible explanation relies in the immune status of penguins that were not neutropenic, on the contrary of the rats of this study. Thus, they were probably more likely to develop anti-*Aspergillus* inflammatory reaction, and this is maybe the particular reason why the interleukin (IL)-17 pathway was particularly solicited in them [38]. However, it is noteworthy to report that three protein pathways were significantly enriched in both species studied, and so we decided to focus on them because they appeared to play a common role during aspergillosis whatever the host: cadherin, Wnt and FGF signaling pathways. Cadherin pathway is involved in cell adhesion by forming *adherens* junctions to bind cells within tissues together [39]. The Wnt signaling pathway is an ancient and evolutionarily conserved pathway that regulates crucial aspects of cell fate determination, cell migration, and cell polarity [40]. Wnt signal stimulates several intra-cellular signal transduction cascades, including the canonical or Wnt/β-catenin dependent pathway and the non-canonical or β-catenin-independent pathway [41]. It was shown that stimulation of Wnt/β-catenin dependent pathway by agonist reduced aspartate aminotransferase, lactate, and lactate dehydrogenase levels by 40%, 36%, and 77% six hours after an hemorrhagic shock generated in Sprague-Dawley rats [42]. Herein, we also confirmed by ELISA assay an overexpression of the Wnt1-inducible-signaling pathway protein 1 (Wisp-1), also known as CCN4 in humans, during experimental aspergillosis. Encoded by the *WISP1* gene, Wisp-1 is a secreted, extracellular matrix-associated signaling protein that is largely involved in the common Wnt pathway [43]. It can attenuate apoptosis and promote tissue growth and fibrosis. In a previous study, neutralizing monoclonal antibodies specific tor Wisp-1 improved both lung function and survival in a model of bleomycin-challenged C57BL/6N mice (+27%; *P*<0.02) [44]. Once activated by secreted proteins of the signaling component of the FGF family, tyrosine kinase FGF receptors are able to phosphorylate specific tyrosine residues that thereafter mediate interaction with cytosolic adaptor proteins and the RAS-MAPK (rat sarcoma—mitogen-activated protein kinase), PI3K-AKT (phosphoinositide 3-kinase–Ak thymoma), PLCγ (phosphoinositide phospholipase Cγ), and STAT (signal transducers and activators of transcription) intracellular signaling pathways [45]. Thus, FGF pathway plays a role during metabolic disorders or in injured tissues, where it mediates metabolic functions, tissue repair, and regeneration, often by reactivating developmental signaling pathways. A few years ago, cocktail therapy combining FGF and Wnt inhibitors, such as small-molecule compounds and human neutralizing antibodies, have been envisaged to increase the efficacy of anti-cancerous chemotherapy through the inhibition of recurrence by destructing cancer stem cells [46]. Besides herein, genome ontology enrichment analysis showed significant over-involvement of blood coagulation cascade in the rat model that means several proteins of this process were significantly overproduced during aspergillosis, perhaps following initial consumption or proteolysis as a consequence of angio-invasion. If we extrapolate this finding to neutropenic humans, one can reasonably speculate this may have consequences on hemorrhagic risks and the occurrence of episodes of disseminated intravascular coagulation, highlighting the need to thoroughly monitor the hemostasis of Hematology patients. Relative increase of several leucine-rich glycoproteins and their derivatives were confirmed by 2.0-fold in rat lungs, 2.6- and 2.0-fold in penguins, as previously reported with 1.4-fold increase in human samples [18]. Although their actual role in promoting host-fungal invasion has not been totally elucidated, leucine-rich glycoproteins have been described as involved in cellular adhesion [47]. Likewise, demonstration of increased secretion of host proteins that control the metabolic processes, like extracellular proteinases and ribonucleotoxin was confirmed herein by our iTRAQ^®^ protocol results [14]: for example, serine peptidase inhibitor clade B member 1b was 3.1-fold overrepresented in rat blood and serine peptidase inhibitor clade A member 3B (alpha-1 antiproteinase, antitrypsin) was 2.1-fold increased in rat lungs. Alpha-1-acid glycoprotein, also referred to as orosomucoid, was measured 2.3-fold increased in infected rat lungs, which was consistent with its *in situ* immune-modulating effects [48]. Expression of the alpha-1-acid glycoprotein gene is controlled by a combination of the major regulatory mediators, *i*.*e*. a pro-inflammatory cytokine network involving mainly IL-1 beta, tumor necrosis factor-alpha, IL-6 and other IL-6 related cytokines, which have been all described in immune response against *Aspergillus* [49]. As interleukin-inhibitors have been proven to be useful in some autoimmune diseases [50], one could consider on the contrary the benefit of drugs stimulating the aforementioned components for the struggle against *Aspergillus* fungus. Surprisingly, inter-alpha-trypsin inhibitor heavy-chain, ITIH4 *i*.*e*. a α1-globulin, was found 2.0-lowered in penguins of this study, whereas it was shown to be approximately 1.7- to 8.5-fold upregulated in *Aspergillus*-diseased rats in our previous work which was based on 2D-gel electrophoresis as pre-MS screening [14]. Regarding specifically the proteins involved in the lipid metabolism, several apolipoproteins have been reported in other models of aspergillosis which is likely related to their well-known antioxidant and anti-inflammatory properties [51]. Herein, apolipoprotein AII and apolipoprotein E were 2.2- and 2.1-fold enriched in diseased rat lungs *vs*. LPS-control lungs, respectively. Both have already been reported 4.0- and 5.8-fold increased in rat BALF [14]. In human blood obtained from leukemic patients with aspergillosis, Brasier *et al*. showed a 1.6-fold decrease of apolipoprotein AII, suggesting a local consumption during tissue invasion [18]. In the present study, eight other proteins related to lipid synthesis or transport were also found with significant changes *vs*. control conditions, like lipid phosphate phosphatase-related protein type 3 in penguins, as well as several phospholipases. For therapeutic purposes, a few short molecules have been recently developed to moderate lipoprotein levels and so they can be valuable to test during aspergillosis [52]. Zinc finger proteins were decreased in both animal species of this study, *i*.*e*. 5.4- and 7.5-fold in rats and penguins, respectively.

Interestingly, our MS findings sometimes contradicted previous studies [18,53]. For example, we failed to prove that the involvement of the individual components of complement cascade was significant during aspergillosis, except for complement C8 in rat lungs (2.5-fold increase *vs*. LPS-control lungs). This finding may indicate that complement components are eventually not so crucial for innate defense against *Aspergillus* and future therapeutic approaches should not only focus on them. Similarly, previous MS approaches defined major changes in various fibrinogen isoforms [18], that have been assumed to play a role in promoting cell adhesion once induced by IL-6 [54]. This hypothesis was not confirmed herein, perhaps because our protein identification was based on very stringent statistics and thus avoided fibrinogen misidentification. Likewise except for ITIH4 protein, [8,22], no significant changes were reported for some specific proteins belonging to α1-, α-2 and β-globulins. This suggests that conventional plasma protein electrophoresis analysis only addresses global changes for each protein moiety [8,55]; the moieties should be considered as the sum of slight–and often insignificant–individual changes [18]).

Overall, use of the iTRAQ^®^ protocol present study provided new insights into host inflammatory response against *Aspergillus* in two relevant infection models. One could just consider these MS findings as preliminary trials for better understanding pathophysiology of aspergillosis, like demonstrated by the common increase of the cadherin, Wnt and FGF signaling pathways. Thus, MS has probably the potential to further refine diagnostics or to suggest therapeutic targets and monitoring markers. Additional studies are now needed to validate these results and expand on the construction of functional protein networks during aspergillosis. This example of iTRAQ^®^ protocol application should be easily reproduced in other species, like humans, and in other diseases.

## Supporting information

S1 Table Summary of the global protein changes observed by iTRAQ^®^ protocol-based mass spectrometry analysis. Only the relative changes that were statistically significant *versus* controls are reported below (for details, see Material & Methods section). Results are expressed in absolute numbers and in percentage for the representative proportion with respect to the total number of proteins identified for each species in GO databases (http://www.geneontology.org/) (between brackets).
(DOCX)

S2 Table Raw data of mass spectrometry analysis. This supplementary material displays the exhaustive listing of all the proteins that have been identified in rats, penguins and humans.
(XLSX)
