# Supplementary material for: Translational proteomic study to address host protein changes during aspergillosis
Source: PLoS One. 2018 Jul 24;13(7):e0200843. doi: 10.1371/journal.pone.0200843 (PMC6057647; doi:10.1371/journal.pone.0200843)
Supplement: S1 Table — Only the relative changes that were statistically significant versus controls are reported below (for details, see Material & Methods section). Results are expressed in absolute numbers and in percentage for the representative proportion with respect to the total number of proteins identified for each species in GO databases (http://www.geneontology.org/) (between brackets). (DOCX) [file pone.0200843.s001.docx]

|  | **Rats** | | **Penguins** |
| --- | --- | --- | --- |
|  | **Blood** | **Lung** | **Blood** |
| **Total number of protein identified** | **7,858** (33.0%) | **4,430** (18.63%) | **6,436** (40.76%) |
| **Number of proteins identified with significant changed levels** | **148** (0.62%) | **325** (1.37%) | **468** (2.96%) |
| **Number of proteins identified with significant increased levels**  [2.0 - 4.0] fold enriched  [4.0 - ∞] fold enriched | **71** (0.30%)  61 (0.26%)  10 (0.04%) | **182** (0.77%)  168 (0.71%)  14 (0.06%) | **171** (1.08%)  154 (0.98%)  17 (0.11%) |
| **Number of proteins identified with significant decreased levels**  [2.0 - 4.0] depleted  [4.0 - ∞] fold depleted | **77** (0.32%)  72 (0.30%)  5 (0.02%) | **143** (0.60%)  124 (0.52%)  19 (0.08%) | **297** (1.88%)  258 (1.63%)  39 (0.25%) |

**S1 Table.** **Summary of the global protein changes observed by iTRAQ^®^ protocol-based mass spectrometry analysis**. Only the relative changes that were statistically significant *versus* controls are reported below (for details, see *Material &* *Methods* section). Results are expressed in absolute numbers and in percentage for the representative proportion with respect to the total number of proteins identified for each species in GO databases (<http://www.geneontology.org/>) (between brackets).
